# Free amino nitrogen concentration correlates to total yeast assimilable nitrogen concentration in apple juice

**DOI:** 10.1002/fsn3.536

**Published:** 2017-11-07

**Authors:** Thomas F. Boudreau, Gregory M. Peck, Sean F. O'Keefe, Amanda C. Stewart

**Affiliations:** ^1^ Department of Food Science and Technology Virginia Polytechnic Institute and State University Blacksburg VA USA; ^2^ School of Integrative Plant Science, Horticulture Section Cornell University Ithaca NY USA

**Keywords:** Apples, cider, fermentation, food processing chemistry, pome fruits

## Abstract

Yeast assimilable nitrogen (YAN) is essential for yeast growth and metabolism during apple (*Malus* x *domestica* Borkh.) cider fermentation. YAN concentration and composition can impact cider fermentation kinetics and the formation of volatile aroma compounds by yeast. The YAN concentration and composition of apples grown in Virginia, USA over the course of two seasons was determined through analysis of both free amino nitrogen (FAN) and ammonium ion concentration. FAN was the largest fraction of YAN, with a mean value of 51 mg N L^−1^ FAN compared to 9 mg N L^−1^ ammonium. Observed YAN values ranged from nine to 249 mg N L^−1^, with a mean value of 59 mg N L^−1^. Ninety‐four percent of all samples analyzed in this study contained <140 mg N L^−1^ YAN, a concentration generally considered the minimum level needed in grape‐based wines for yeast to fully utilize all of the fermentable sugars. FAN concentration was correlated with total YAN concentration, but ammonium concentration was not. Likewise, there was no correlation between FAN and ammonium concentration.

## 1 INTRODUCTION

Yeast assimilable nitrogen (YAN) is an essential nutrient for yeast growth and metabolism during fruit juice fermentation. YAN is composed of ammonium ions and free amino nitrogen (FAN). The relative composition of amino acids making up the amino nitrogen portion varies across fruit species and cultivar (Ma, unpublished). Yeast metabolism is also influenced by the interaction between yeast strain (Julien, Roustan, Dulau, & Sablayrolles, 2000) and the chemical composition of YAN (Torrea et al., 2011). Ammonium is preferentially assimilated by yeasts (Jiranek, Langridge, & Henschke, 1995a) and can be the sole YAN source used to complete fermentation (ter Schure, van Riel, & Verrips, 2000). However, FAN can lead to higher maximum fermentation rates when present in combination with ammonium (Beltran, Esteve‐Zarzoso, Rozes, Mas, & Guillamon, 2005). Similarly, fermentations with high FAN concentrations lead to desirable flavor production in grape‐based wines as compared to ammonium alone (Torrea et al., 2011).

In grape‐based wine (*Vitis vinifera*) production, a commonly accepted minimum YAN concentration is 140 mg N L^−1^ (Bely, Sablayrolles, & Barre, 1990). However, minimum YAN concentrations as high as 267 mg N L^−1^ (Mendes‐Ferreira, Mendes Faia, & Leão, 2004) or even 350 mg N L^−1^ have been suggested for juices containing soluble solid concentration over 24° Brix (Bisson & Butzke, 2000). In wine grape fermentations, YAN deficiencies have been demonstrated to slow fermentation rates (often referred to as “sluggish fermentations”) or to result in incomplete sugar utilization (often referred to as a “stuck fermentation”) (Bisson, 1999; Blateyron & Sablayrolles, 2001; Ingledew & Kunkee, 1985). This is partly attributable to insufficient YAN concentrations resulting in lower yeast biomass (Martínez‐Moreno, Morales, Gonzalez, Mas, & Beltran, 2012; Varela, Pizarro, & Agosin, 2004).

Low YAN concentration also contributes to increased production of hydrogen sulfide, a compound widely associated with unpleasant aromas and decreased consumer acceptance (Jiranek, Langridge, & Henschke, 1995b; Ugliano, Kolouchova, & Henschke, 2011; Vos, Vos, & Gray, 1979). Low YAN concentration has also been associated with production of other undesirable compounds, such as higher alcohols, and with the lack of formation of positive flavor compounds, such as esters and volatile fatty acids (Bell & Henschke, 2005). Therefore, wines made from YAN deficient grape juice can have lower sensory ratings compared to wines made from juice with sufficient YAN concentration (Ugliano, Travis, Francis, & Henschke, 2010).

Although extensive research has been conducted on the YAN concentration and composition of grape juice and its influence on wine fermentation, there is limited information available on apple YAN concentration and composition, or how those factors affect cider fermentation. There is limited research on how pre‐harvest factors affect apple nitrogen content, but in winemaking many factors are known to contribute to final fruit nitrogen, including soil and foliar nitrogen fertilization, rootstock genetics, and crop load (Bell & Henschke, 2005). One study found that increasing apple crop load led to decreased apple YAN (Peck, McGuire, Boudreau, & Stewart, 2016). Another study found amino acids to decrease during fruit ripening (Mangas, Moreno, Picinelli, & Blanco, 1998). Alberti, Vieira, Drilleau, Wosiacki, & Nogueira, 2011 found that the average FAN concentration of 51 apple samples in Brazil was 38.3 mg N L^−1^, but YAN was not quantified. Yeast assimilable nitrogen levels in cider apples grown in New York State ranged from 12 to 190 mg N L^−1^, but the relative contributions of FAN and ammonium ions were not reported in that study (Valois, Merwin, & Padilla‐Zakour, 2006). Currently, there are no reports of YAN concentration and composition for apples grown in other production regions of the U.S. An improved understanding of endogenous apple YAN concentration is of particular interest to apple growers and cider producers, as this parameter can be used to inform and optimize cider fermentation practices. In the United States, cider production has increased by nearly nine‐fold over the last decade, creating an immediate need for research into practices that maximize cider quality (Peck & Miles, 2015).

The objective of this study was to determine YAN, FAN, and ammonium concentrations of apple cultivars used for cider production in Virginia and examine whether correlations between those parameters exist.

## 2 MATERIALS AND METHODS

### 2.1 Juice samples

In 2014 and 2015, apple samples from 12 cultivars were collected from research orchards at the Virginia Tech Alson H. Smith, Jr. Agricultural Research and Extension Center near Winchester, VA. Arkansas Black, Blacktwig, Field Red, Granny Smith, Newtown Pippin (syn. Albemarle Pippin), Northern Spy, Winesap, and York were grafted onto MM.111 rootstock and planted as single trees in 1983 as part of a heritage display planting. The remaining four cultivars were Empire grafted onto MARK rootstock planted in 1991, Enterprise grafted onto M.9 rootstock planted in 1995, Golden Delicious grafted onto M.9 rootstock planted in 2000, and Virginia Gold grafted onto M.9 rootstock planted in 2011. All trees received annual dormant pruning and standard pest control as per regional recommendations (Pfeiffer et al., 2010). None of the trees had received nitrogen fertilizer for at least 3 years prior to the start of this experiment. Thirty apples were harvested at commercial maturity as determined by a starch pattern index rating of at least five on a scale of one to eight (Blanpied & Silsby, 1992) from a height between 1–2 m from the ground and from all sides of the exterior canopy of each sample tree in each year. Overall, the 2014 growing season was relatively mild, with only a few days above 35°C and no extended periods of drought. The 2015 season had similarly mild temperatures, but precipitation was 44% and 58% below the long‐term mean during July and August, respectively. Fruit maturity data for all cultivars for 2014 and 2015 are presented in Tables S1 and S2 The 30 apples were randomly separated into three groups of 10 apples each and all downstream processing occurred separately for each subsample to generate analytical replicates. Apples were juiced (Champion Juicer 2000, Lodi, CA, USA) the same day they were harvested and juice was stored at −20° C in 15 ml centrifuge tubes until the time of analysis.

### 2.2 Analytical methods

YAN was quantified in the Enology and Fermentation Lab in Blacksburg, VA using assays for free amino nitrogen (K‐PANOPA kit, Megazyme, Wicklow, Ireland) and ammonium ion (Ammonia‐Rapid kit, Megazyme, Wicklow, Ireland). Samples were thawed to 22°C and centrifuged at 1,096 × g for 5 min prior to analysis. Data were subjected to a t‐test with significance defined as *p* < .05 followed by parametric mean testing using Tukey's Honestly Significant Difference (HSD) test (GraphPad Prims v.6, La Jolla, CA, USA). Regression analysis was used to determine correlation coefficients (*p* < .05).

## 3 RESULTS

**Figure 1:**
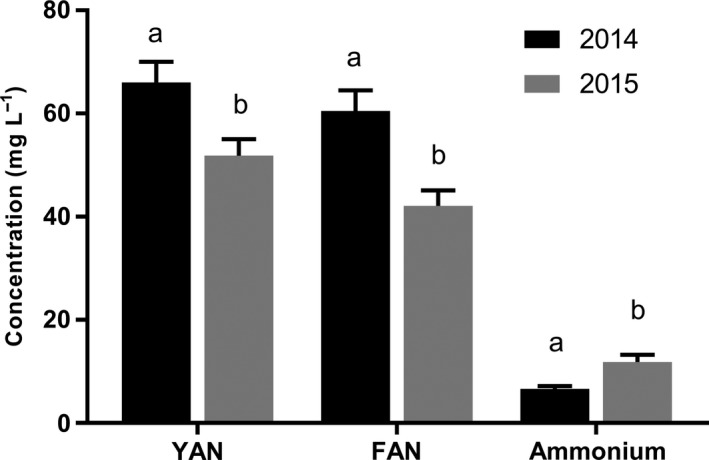
Comparison of average yeast assimilable nitrogen (YAN), free amino nitrogen (FAN) and ammonium concentrations observed in the 2014 and 2015 seasons (*n*=108 total apple samples). Error bars signify standard error of the mean, and different lowercase letters indicate differences in a given value between the 2014 and 2015 seasons (two‐way t‐test with significance defined as p<0.05)

YAN concentration, FAN concentration and ammonium concentration all differed significantly between the 2014 and 2015 seasons (*p* < .05) (Figure 1). YAN concentration for all samples in both years averaged 59 ± 3 mg N L^−1^. Mean overall FAN concentration was 51 ± 3 mg N L^−1^ and mean overall ammonium concentration was 8 ± 1 mg L^−1^ (Figure 1). While total YAN concentration and FAN concentration decreased from 2014 to 2015, ammonium concentration increased from 2014 to 2015 (Figure 1). For all samples in both years, FAN comprised the largest proportion of apple juice YAN. On average FAN comprised 85% of total YAN, while ammonium accounted for the other 15% (Figure 1).

**Figure 2:**
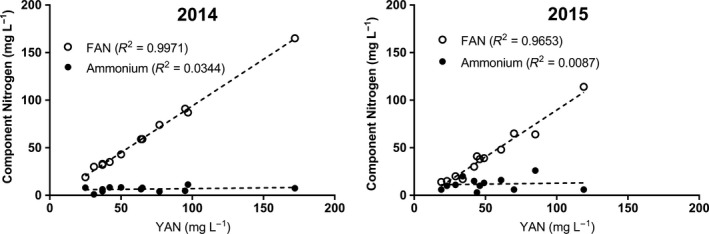
Linear regressions and correlation coefficients of free amino nitrogen (FAN) versus yeast assimilable nitrogen (YAN) and ammonium versus YAN for apples harvested in 2014 and 2015

**Figure 3:**
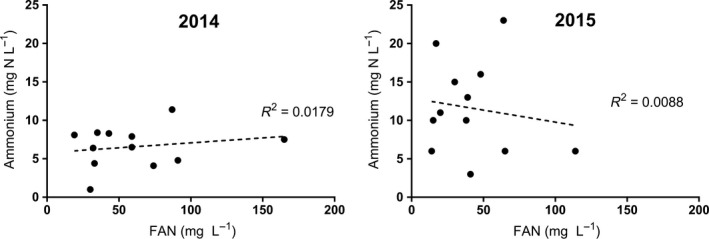
Linear regressions and correlation coefficients of ammonium and free amino nitrogen (FAN) concentration for apples harvested in 2014 and 2015

FAN concentration was highly correlated (*p* < .0001) with total YAN in both 2014 (*R* ^2^ = 0.9971) and 2015 (*R* ^2^ = 0.9653) (Figure 2). However, there was no correlation between ammonium concentration and total YAN in 2014 (*p* = .4295) or 2015 (*p* = .7463) (Figure 2) or between ammonium and FAN concentration in 2014 (*p* = .5337) or 2015 (*p* = .7850) (Figure 3).

## 4 DISCUSSION

The majority of the samples in this study contained <140 mg N L^−1^, a value often considered the minimum recommended YAN concentration for fermentation of fruit juice by *Saccharomyces cerevisiae* (Bell & Henschke, 2005; Bely et al., 1990). The minimum YAN required specifically for cider fermentation has not been established, but research‐based recommendations for wine fermentation are currently being used by many cider makers as a baseline for cider fermentation. One study conducted in model grape juice suggested that an even higher minimum concentration of 267 mg N L^−1^ is needed to result in complete fermentation with starting concentration of 20 g L^−1^ glucose (Mendes‐Ferreira et al., 2004). This value is well above the average YAN concentrations found in apple cultivars in this study. Prior research has indicated that the amount of YAN required for wine fermentation is correlated with the concentration of soluble solids present in the fermenting medium, where higher soluble solids concentration requires a higher YAN concentration to complete fermentation (Bisson, 1999). One study found that fermentation ended before all sugar had been consumed at initial nitrogen concentrations below 153 mg L^−1^ (Mendes‐Ferreira et al., 2004). The fact that cider fermentations are conducted with lower soluble solids concentration than wine fermentations has led some in the cider industry to hypothesize that the YAN concentration required for apple fermentations could be lower than those required for grape juice fermentation. However, the many factors interacting with total YAN concentration to impact fermentation make extrapolation of these findings into a cider fermentation system inadvisable (Boudreau, Peck, O'Keefe, & Stewart, 2017b; Boudreau et al., 2017a). Pre‐fermentation juice clarification methods also differ among wine and cider producers, with multiple clarification methods being employed at the commercial scale for both applications. Significant differences in YAN concentration have been reported for enzymatically clarified apple juice, but not for centrifuged juice (Ma, 2016).

Currently, there is very limited information available on YAN concentration and composition for apple juice being used for cider production, although this parameter plays an important role in effective fermentation management. One study quantified total nitrogen using the Kjeldahl method, which was found to be 155.8 mg N L^−1^ (Alberti et al., 2011), however this value has limited significance in relation to beverage fermentation as it includes proline and nitrogen present in protein and other forms unavailable for yeast metabolism (Bell & Henschke, 2005). Our study provides new and useful information on the YAN concentration and composition of several apple cultivars that are currently being used for cider making.

Total YAN concentration decreased from 2014 to 2015, on average (Figure 1). However, within the cultivars examined in this study, this trend was not consistent. For some cultivars, YAN increased from 2014 to 2015, while for others it decreased. While the *t*‐test used to compare YAN between the two growing seasons in this study may, to a degree, conflate multiple sources of variation (e.g., rainfall, temperature means, length of growing season in each year), the practical implications merit consideration. This finding demonstrates that apple juice with sufficient YAN concentration for fermentation in 1 year may not have sufficient YAN the next year, even for the same cultivar grown using similar management practices in both seasons. YAN requirements and concentration in fruit can also be influenced by other production system related factors such as differences in fungicide residues (Boudreau et al., 2017b) and crop load (Peck et al., 2016).

Ammonium concentrations did not exceed 9 mg L^−1^ on average in either year of our study. Slower fermentation rates due to limited YAN may be exacerbated by the limited ammonium concentrations found in apples compared to the higher proportion of YAN made up by ammonia in grape systems. Ammonium is consumed preferentially by yeast during wine fermentation (Jiranek et al., 1995a; ter Schure et al., 2000), and FAN consumption can decline dramatically or be completely inhibited in the presence of ammonium in the fermenting medium (Jiranek et al., 1995a). Low ammonium concentrations in apples in combination with low overall total YAN may contribute to sluggish fermentations and the formation of undesirable aroma compounds. Grape juice for wine production generally contains higher concentrations of both total YAN and ammonium ions than we currently report in apple juice. Thus, the findings of grape juice‐based fermentation (wine) should not be extrapolated to apple juice‐based fermentation (cider). The effects of the relatively low concentrations of YAN and ammonium ions observed in this study on cider fermentation performance warrant further investigation.

In both years investigated in this study, there was a strong correlation between FAN concentration and YAN concentration observed within each cultivar analyzed (Figure 2). This observed effect was due to the low concentrations of ammonium observed in this study, making FAN the largest portion of the total YAN. This finding suggests that orchard managers and cider makers may be able to accurately estimate total YAN by measuring FAN alone, lowering costs and time associated with laboratory analyses of both FAN and ammonium. Measurement of both FAN and ammonium is required for YAN determination in wine grapes, as there is no correlation between grape FAN and YAN, or grape ammonium ion concentration and YAN (Butzke, 1998). Further research in additional growing regions and cultivars will be necessary to determine whether FAN measurement alone can be reliably used to proportionally quantify total YAN in apple juice, and whether the concentration of ammonium, even when very low compared to total YAN, affects fermentation performance and resulting cider quality. There was no correlation between ammonium and FAN concentration in either year within cultivars examined in this study (Figure 3). This observation is similar to previous findings reported in surveys of YAN concentration and composition in grape juice (Butzke, 1998). For cider makers in the Eastern United States, this indicates that there may be large variations in FAN and ammonium concentration across cultivar and growing seasons, further emphasizing the need to measure YAN seasonally and for each cultivar and orchard block or juice blend for accurate total YAN quantification pre‐fermentation.

## 5 CONCLUSION

Our analysis indicates that apple juice is often YAN deficient for fermentation, by current wine industry recommendations. There was a large variation in YAN concentration among the samples evaluated in this study. Furthermore, YAN concentration varied significantly across growing season for most cultivars investigated, even with fruit harvested from the same trees grown using similar management practices in both years. Cider makers in Eastern United States should be aware of the potential for YAN deficiency, and take appropriate action to measure YAN pre‐fermentation and supplement juice with appropriate nitrogen adjuncts to augment YAN prior to fermentation if deemed necessary. A correlation between FAN and total juice YAN was observed in this study. This finding indicates that cider makers could potentially measure only FAN and still accurately quantify total YAN in apple juice, unlike grape juice which requires separate determination of both components of YAN. Further evaluation will be required to determine the extent to which this strategy can be applied across apple cultivars and growing regions.
