# Supplementary material for: Free amino nitrogen concentration correlates to total yeast assimilable nitrogen concentration in apple juice
Source: Food Sci Nutr. 2017 Nov 7;6(1):119–23. doi: 10.1002/fsn3.536 (PMC5778214; doi:10.1002/fsn3.536)
Supplement: Supplementary file 1 [file FSN3-6-119-s001.docx]

| **Table S1** – Physical and Chemical Parameters of Apples Harvested in 2014 ^a^ | | | | | | | | | | | |
| --- | --- | --- | --- | --- | --- | --- | --- | --- | --- | --- | --- |
|  | **Mass (g)** | **Diameter (mm)** | **Flesh Firmness (N)** | **Soluble Solids (°Brix)** | **TA (g/L)** | **pH** | **Ethylene (mL/L)** | **YAN (mg/L)** | | **FAN (mg/L)** | **Ammonia (mg/L)** |
| Albemarle Pippin | 178.7 ± 4.3 | 75.2 ± 0.2 | 85.1 ± 0.3 | 11.5 ± 0.6 | 6.04 ± 0.25 | 3.40 ± 0.01 | 0.0 ± 0.0 | 77 ± 4 | 74 ± 3 | | 3 ± 1 |
| Arkansas Black | 205.5 ± 6.2 | 77.7 ± 0.5 | 114.4 ± 1.3 | 9.4 ± 0.5 | 5.07 ± 0.14 | 3.55 ± 0.01 | 0.0 ± 0.0 | 64 ± 4 | 59 ± 3 | | 5 ± 2 |
| Blacktwig | 190.0 ± 0.8 | 77.9 ± 0.3 | 106.4 ± 0.5 | 10.2 ± 1.0 | 6.21 ± 0.63 | 3.22 ± 0.05 | 0.0 ± 0.0 | 42 ± 3 | 35 ± 3 | | 7 ± 2 |
| Empire | 163.1 ± 1.4 | 74.1 ± 0.2 | 63.7 ± 0.5 | 8.9 ± 0.8 | 4.21 ± 0.26 | 3.15 ± 0.18 | 92.5 ± 10.2 | 95 ± 7 | 91 ± 7 | | 4 ± 1 |
| Enterprise | 286.1 ± 5.2 | 88.1 ± 0.8 | 82.8 ± 0.8 | 12.3 ± 0.3 | 6.85 ± 0.56 | 3.40 ± 0.01 | 8.8 ± 1.1 | 172 ± 12 | 165 ± 13 | | 6 ± 2 |
| Field Red | 130.6 ± 2.5 | 69.1 ± 0.5 | 111.9 ± 0.9 | 11.3 ± 0.4 | 5.22 ± 0.07 | 3.45 ± 0.02 | 3.2 ± 2.4 | 31 ± 2 | 30 ± 2 | | 1 ± 1 |
| Golden Delicious | 165.9 ± 7.2 | 72.6 ± 1.1 | 67.2 ± 1.3 | 9.6 ± 0.9 | 3.48 ± 0.45 | 3.55 ± 0.01 | 0.1 ± 0.0 | 25 ± 2 | 19 ± 1 | | 7 ± 1 |
| Granny Smith | 198.8 ± 5.0 | 77.6 ± 0.7 | 71.7 ± 0.8 | 11.7 ± 0.2 | 4.21 ± 0.13 | 3.32 ± 0.05 | 0.0 ± 0.0 | 97 ± 5 | 87 ± 5 | | 9 ± 2 |
| Northern Spy | 234.1 ± 5.6 | 85.6 ± 0.7 | 72.1 ± 0.5 | 9.9 ± 0.5 | 6.58 ± 0.22 | 3.30 ± 0.04 | 115.8 ± 13.4 | 37 ± 2 | 33 ± 2 | | 4 ± 2 |
| Old York | 172.2 ± 5.5 | 77.0 ± 0.5 | 82.3 ± 1.2 | 8.5 ± 0.4 | 4.81 ± 0.49 | 3.51 ± 0.00 | 0.3 ± 0.3 | 65 ± 4 | 59 ± 3 | | 7 ± 1 |
| Virginia Gold | 252.2 ± 3.3 | 83.4 ± 0.2 | 68.0 ± 0.7 | 9.2 ± 0.5 | 6.68 ± 0.24 | 3.39 ± 0.00 | 0.0 ± 0.0 | 37 ± 1 | 32 ± 2 | | 5 ± 2 |
| Winesap | 133.5 ± 3.3 | 69.3 ± 0.7 | 84.6 ± 0.2 | 11.4 ± 0.4 | 5.03 ± 0.26 | 3.52 ± 0.01 | 0.0 ± 0.0 | 50 ± 4 | 43 ± 4 | | 7 ± 1 |
| ^a^ values expressed as average ± standard error | | | | | | | | | | | |
