# Supplementary material for: Free amino nitrogen concentration correlates to total yeast assimilable nitrogen concentration in apple juice
Source: Food Sci Nutr. 2017 Nov 7;6(1):119–23. doi: 10.1002/fsn3.536 (PMC5778214; doi:10.1002/fsn3.536)
Supplement: Supplementary file 2 [file FSN3-6-119-s002.docx]

| **Table S2** – Physical and Chemical Parameters of Apples Harvested in 2015 ^a^ | | | | | | | | | | |
| --- | --- | --- | --- | --- | --- | --- | --- | --- | --- | --- |
|  | **Mass (g)** | **Diameter (mm)** | **Flesh Firmness (N)** | **Soluble Solids (°Brix)** | **TA (g/L)** | **pH** | **Ethylene (mL/L)** | **YAN (mg/L)** | **FAN (mg/L)** | **Ammonia (mg/L)** |
| Albemarle Pippin | 148.8 ± 8.4 | 70.6 ± 1.0 | 97.4 ± 0.5 | 9.2 ± 0.7 | 8.76 ± 0.08 | 3.38 ± 0.08 | 1.0 ± 0.6 | 85 ± 11 | 64 ± 6 | 22 ± 10 |
| Arkansas Black | 184.8 ± 8.9 | 74.6 ± 1.1 | 109.1 ± 1.1 | 9.1 ± 0.2 | 5.94 ± 0.09 | 3.68 ± 0.01 | 1.6 ± 0.2 | 46 ± 4 | 38 ± 4 | 8 ± 2 |
| Blacktwig | 199.8 ± 3.1 | 78.6 ± 0.2 | 90.5 ± 0.5 | 10.2 ± 0.4 | 6.10 ± 0.25 | 3.35 ± 0.04 | 23.6 ± 7.9 | 19 ± 2 | 14 ± 2 | 5 ± 1 |
| Empire | 160.3 ± 1.1 | 72.1 ± 0.1 | 73.4 ± 0.2 | 8.4 ± 0.2 | 7.15 ± 0.36 | 3.17 ± 0.12 | 1.6 ± 0.8 | 34 ± 5 | 17 ± 3 | 17 ± 3 |
| Enterprise | 174.3 ± 10.2 | 73.5 ± 1.6 | 82.1 ± 0.1 | 8.7 ± 0.5 | 6.44 ± 0.04 | 3.23 ± 0.01 | 1.7 ± 0.8 | 119 ± 13 | 114 ± 12 | 5 ± 3 |
| Field Red | 137.7 ± 2.2 | 69.9 ± 0.5 | 108.2 ± 2.9 | 12.1 ± 0.4 | 7.48 ± 0.41 | 3.04 ± 0.05 | 3.2 ± 1.5 | 23 ± 3 | 15 ± 2 | 8 ± 2 |
| Golden Delicious | 236.7 ± 3.0 | 81.6 ± 0.5 | 62.7 ± 1.2 | 8.4 ± 0.5 | 3.89 ± 0.06 | 3.37 ± 0.09 | 3.1 ± 0.2 | 70 ± 4 | 65 ± 3 | 5 ± 2 |
| Granny Smith | 219.9 ± 6.6 | 79.9 ± 0.8 | 72.6 ± 0.1 | 8.8 ± 0.4 | 6.82 ± 0.27 | 3.50 ± 0.03 | 8.0 ± 2.0 | 29 ± 5 | 20 ± 3 | 9 ± 3 |
| Northern Spy | 175.5 ± 10.1 | 76.5 ± 1.3 | 79.0 ± 1.0 | 8.5 ± 0.6 | 7.80 ± 0.84 | 3.08 ± 0.23 | 27.4 ± 1.4 | 61 ± 5 | 48 ± 5 | 13 ± 3 |
| Old York | 170.5 ± 4.6 | 75.2 ± 1.1 | 91.0 ± 1.7 | 9.1 ± 0.2 | 5.49 ± 0.08 | 3.58 ± 0.06 | 2.4 ± 1.1 | 49 ± 8 | 39 ± 5 | 11 ± 5 |
| Virginia Gold | 186.8 ± 6.8 | 75.7 ± 0.8 | 75.1 ± 0.8 | 8.2 ± 0.2 | 8.13 ± 0.45 | 3.19 ± 0.24 | 4.9 ± 0.4 | 44 ± 3 | 41 ± 2 | 3 ± 1 |
| Winesap | 138.3 ± 1.4 | 68.7 ± 0.3 | 98.0 ± 0.7 | 9.9 ± 0.7 | 7.02 ± 0.35 | 3.46 ± 0.13 | 2.7 ± 0.8 | 42 ± 5 | 30 ± 3 | 12 ± 5 |
| ^a^ values expressed as average ± standard error | | | | | | | | | | |
